# Aberrant lipid metabolism in cancer cells and tumor microenvironment: the player rather than bystander in cancer progression and metastasis

**DOI:** 10.7150/jca.64833

**Published:** 2021-11-04

**Authors:** Xiujing Yu, Shuyi Mi, Jun Ye, Guochun Lou

**Affiliations:** 1 Department of Endoscopy Center, The Second Affiliated Hospital of Zhejiang University School of Medicine, Hangzhou, Zhejiang Province, China.; 2 Department of Gastroenterology, The Second Affiliated Hospital, Zhejiang University School of Medicine, Hangzhou, Zhejiang Province, China.

**Keywords:** lipid metabolism remodeling, tumor microenvironment, cancer metastasis, intrinsic factors, extrinsic factors

## Abstract

As the primary cause of cancer-induced fatality and morbidity, cancer metastasis has been a hard nut to crack. Existing studies indicate that lipid metabolism reprogramming occurring in cancer cells and surrounding cells in TME also endows the aggressive and spreading properties with malignant cells. In this review we describe the lipid metabolic reprogramming of cancer cells at different steps along the metastatic process, we also summarize the altered lipid metabolism of non-cancer cells in TME during tumor metastasis. Additionally, we reveal both intrinsic and extrinsic factors which influence the cellular lipid metabolism reprogramming.

## Introduction

As the primary cause of cancer-induced fatality and morbidity, cancer metastasis has been a hard nut to crack 1. It involves a complex process by which a specific subgroup of cancer cells detaches from the primary tumor and colonizes into a non-local environment 2. Recently, it has been suggested that cancer cells are able to adjust their metabolism selectively at different steps along the metastatic cascade, indicating that metabolism is not just a bystander, but it is a necessary part of the process of metastasis 3.

Reprogramming of cellular metabolism has been considered as a major hallmark of cancer, it fuels fast cell growth as well as provides tumor cells with several benefits, including macromolecule biosynthesis, adaptation to the microenvironment, and an ability to cope with oxidative stress 4-6. Warburg effect is a kind of metabolism rewiring that cancer cells shift from oxidative phosphorylation to aerobic glycolysis and many studies have reported Warburg effect plays a significant role in cancer metastasis 7, 8. Relatively, lipid metabolism dysregulation in cancer cells has received less concern but is gradually recognized one of the significant characteristics of cancer cell metabolism which contributes to the progression of various cancers such as colorectal cancer (CRC) and hepatocellular cancer (HCC) 9, 10.

Lipids, also known as fats, contain thousands of different types of molecules, including phospholipids, fatty acids (FAs), triglycerides, sphingolipids, cholesterol and cholesteryl easters (CE) 11. Besides their role as energy storage and critical components of all membranes, lipids could serve as mediators of cancer-relevant phenotypes that promote transformation and tumor growth 12. Increasing numbers of studies have provided evidence that the altered lipid metabolism in malignant cells is capable of being benefit to cancer cell migration, invasion and metastasis 13. However, the precise role of lipid metabolism remodeling in tumor metastasis and the direct mechanistic link between the two still remains to be clarified.

It is also worth noting that in addition to cancer cells, the surrounding cells in tumor microenvironment (TME) such as fibroblasts, immune cells and adipocytes may undergo lipid metabolism reprogramming as well 14-16. The metabolic cooperativity among cancer cells or between cancer cells and stromal cells are likely to benefit cancer cell fitness in anoxic, nutrient-deficient, acidic microenvironment, which promotes the metastasis finally 17. Also, as the common condition of TME, acidosis and hypoxia play a part in the control of lipid anabolism and catabolism via transcriptional regulation 18, 19. On this basis, understanding the factors introducing the lipid metabolism remodeling may help find out new auxiliary therapeutic targets for cancer. Alterations in oncogenes as internal factors directly lead to cellular metabolism modulation, which is important for primary cancer cells to metastasize 20. It is also worth noting that systemic metabolism and gastrointestinal microbiota are able to serve as extrinsic factors to regulate many metabolic processes in the host, which may finally have an effect on cellular metabolism remodeling 21, 22.

The association between lipid metabolism reprogramming and cancer development has been well established, but the global lipid metabolism reprogramming in cancer metastasis still remains elusive. Therefore, in this review we describe the altered lipid metabolism of cancer cells occurring in cancer metastasis and unravel the underlying mechanisms. We also summarize the altered lipid metabolism occurring in TME which may cooperate with cancer cells to induce progression of cancer. In addition, the possible regulations of both extrinsic and intrinsic factors on lipid metabolism remodeling in cancer cells are also discussed in this paper. We believe that revealing lipid metabolism reprogramming in cancer cells and TME will provide more opportunities for clinically directing against cancer metastasis.

## Altered lipid metabolism in cancer cells occurring in metastasis

The altered lipid metabolism in cancer cells involves a series of altered enzyme activities and were considered to be associated with the metastasis of various cancers to a certain extent 3, 11. For instance, the main enzymes participating in *de novo* lipogenesis include fatty acid synthase (FASN), ATP citrate lyase (ACLY) and Acetyl-CoA Carboxylase (ACC), and studies have shown that the altered expression levels of these enzymes are closely related to increased migration and invasion of cancer cells in various cancers, such as pancreatic cancer, breast cancer (BC), HCC and so on 23-26. Also, it is reported that FASN which is highly expressed in primary CRC and liver metastases are capable of upregulating expression of a transmembrane glycoprotein implicated in cancer metastasis called CD44, thus exerts a critical effect on CRC metastasis 27. Among the proteins participated in FAs uptake, CD36 is a dominating player in metabolic tissues which increases long chain fatty acids uptake and has a unique role of initiating the metastasis in many types of cancers 28. The key enzyme for the fatty acid oxidation (FAO) pathway carnitine palmitoyl transferase (CPT), the abnormal expression of which has been found to be associated with cancer cells proliferation in BC, prostate cancer (PC), lung cancer and so on 29. Inside cells, acyl-CoA cholesterol acyltransferase (ACAT) is able to esterify excess free cholesterol into CE and store it in lipid droplets (LDs), which results in high metabolic activity and avoidance of lipotoxicity from free cholesterol 30. Additionally, accumulating CE via ACAT-1 are found to be linked with metastasis in PC, for CE may keep signaling pathways active by maintaining a low free cholesterol environment 31.

Known as the first step of metastasis, epithelial-to-mesenchymal transition (EMT) which is characterized by losing epithelial markers together with enhanced mesenchymal markers confers to cancer cells increased survival, stemness and metastasis-initiation ability 32. It has been reported that phosphorylation-dependent ACC inhibition is a common but critical event in EMT of human solid carcinomas 25. Cellular acetyl-CoA levels are significantly upregulated due to the inhibition of ACC and directly promote acetylation and nuclear translocation of Smad transcription factor to mediate EMT 33. In salivary adenoid cystic carcinoma (SACC), the mesenchymal cancer cells were found to secrete more free fatty acids (FFAs) at the invasive front. While the accumulation of FFAs significantly boosts Src and matrix metalloproteinases (MMP) 9 expression, both of which induce an increase of Stat5‐DNA to enable tumor migration and invasion in mouse model 34. In CRC, it has been demonstrated that upregulation of *de novo* lipogenesis plays a crucial role in rendering the capabilities of adhesion, migration and invasion to CRC cells via upregulation of sphingolipid metabolism 35. Also, inhibition of FASN and sphingolipid metabolism significantly decreases tumor markers associated with adhesion and migration such as pMET, pFAK, PAX in mouse model 35.

Conceivably, after acquiring the capability of migration, metastasizing cancer cells must constantly adapt their metabolism to the distinct environment of the blood or lymph based on the nutrients available in corresponding microenvironment as long as they leave the primary tumor site 20, 36. It was reported that the initial step of various cancers metastasis is more likely to occur though LN which may not be required for metastasis through the blood 37. Actually, LN-metastatic tumors exhibit higher accumulation of FAs as fuel in the lipid-rich LN niche and adapted cells prefer to depend on FAO rather than glucose or glutamine oxidation as a main pathway for generating energy 38. As for metastasizing cancer cells in circulation, it is supposed that FA metabolism is also capable of supporting the survival of circulating cancer cells for exogenous lipids could be used and metabolized by these cells 3. It has been confirmed that oxidative stress induced by reactive oxygen species (ROS) is proved to be harmful to circulating cancer cells *in vivo* 39. As the key enzyme of the FAO pathway, CPT1A plays a critical role in generating cellular NADPH to eliminate ROS by activating FAO pathway, thus grants the detached cancer cells with ability of anoikis resistance. Suppressing CPT1A by etomoxir was demonstrated to decrease metastatic formation *in vivo*, which provides a potential target for cancer metastatic treatment 40.

Finally, these metastasizing cancer cells extravasate and colonize into a new organ to complete the metastasis 41. It was supposed that cancer stem cell (CSC) or metastasis-initiating cell (MIC) which owns ability of maintaining self-renewal and proliferation plays an indispensable role in this final step of metastasis 33. When it comes to MICs, it is worth mentioning cancer cells overexpressing CD36 which are able to initiate metastasis with recapitulation of their molecular and cellular heterogeneity from the primary origin 42. Although CD36^+^ cells have the function of lipid uptake, a recent study has shown that their potential for initiating cancer metastasis requires the involvement of CD36-induced FAO activation 43. When inhibiting the function of CD36, the endogenously synthesized, unmetabolized lipids accumulate continuously in cancer cells, finally leads to metastatic lipotoxicity and cell death 43.

## The lipidomic reprogramming of surrounding cells in TME

Currently, TME become to be believed as an arena where tumor cells constantly interact with various microenvironmental components, giving rise to a protean landscape in which the tumor cells, the host cells and other tumor-associated cells acquire phenotypic alterations 44. Cancer cells are able to bypass the bloodstream and acquire nutrients by scavenging macromolecules from TME 45. Therefore, the microenvironment becomes hypoxic when vasculatures are inadequate or cancer cells are highly proliferating 46. Apart from cancer cells, TME is populated by highly heterogeneous groups of cells, such as cancer-associated fibroblasts (CAFs), tumor-associated macrophages (TAMs), endothelial cells, adipose cells, myeloid-derived suppressor cells (MDSCs), and other immune and inflammatory cells 47. In this case, cancer cells may compete or cooperate with other cells in TME for nutrients, and other cells, like cancer cells, will undergo a similar lipid metabolic remodeling to a certain extent to meet the needs of their own growth just like cancer cells, ultimately promoting metastasis and increasing invasiveness 17.

It has become clear that various kinds of immune cells accelerate metastasis, partially due to their involvement of the establishment of an immunosuppressive microenvironment within primary lesions 48. Emerging studies have supposed that TAMs are the key cells in promoting cancer metastasis by suppressing tumor immune surveillance 49, 50. It was discovered that in a fatty acid-enriched niche induced by cancer cells, lipid droplets (LDs) originated FAs polarize the infiltrating monocytes into M2-like pro-tumoral macrophages, facilitating cancer escape from immune surveillance 49. Compared with control macrophages, TAMs tended to have a higher level of lipid uptake and accumulation via CD36, which accordingly promotes the FAO of TAMs to produce more energy 51. Additionally, as a response to signals from tumor cells in TME, TAMs upregulate the lipid biosynthesis to generate more ROS and produce higher levels of extracellular cytokines like interleukin-6 (IL-6), tumor necrosis factor-α (TNF-α) and so on, which ultimately promotes cancer cell survival, metastasis, angiogenesis and immune suppression 52. Consistently, it has been found that increased lipid biosynthesis in TAMs contribute to the production of inflammatory phenotype and ROS production which are correlated with their protumoral functions, while inhibiting key enzymes of lipid biosynthesis in the TAMs could reverse the enhanced inflammatory cytokines and ROS, reducing the capacity of promoting cancer progression 51.

As a kind of specialized fibroblasts, CAFs are considered to be the principal non-cancerous cell type within TME, which actively promotes tumor cell differentiation and support metastasis by promoting matrix remodeling and EMT 53. In order to fit into the deficiency of nutrients and oxygen in TME, CAFs undergo metabolic reprogramming 54. A study has revealed enhanced fatty acid synthesis and reduced catabolism in CAFs of CRC tissues, and CAFs are able to secret FAs that is took up by CRC cells for the synthesis of other lipids to accelerate the migration of CRC cells, which suggests there possibly exists a novel lipidomic interaction between CAFs and cancer cells 55. In addition, it has been demonstrated that under the influence of CAFs, which have a higher capacity of synthesizing lipids, breast cancer cells are inclined to uptake rather than synthesize lipids, thus increasing the accumulation lipids 56. It is also worth mentioning that CAFs in colon cancers enhance the expression of CPT-1A to actively oxidize FAs, thus enhancing the ability of CAFs to secrete cytokines such as CCL2, VEGF-A, and MMP2 which are associated with angiogenesis and metastasis of tumor cells. Recent investigation was found that blocking FAO in CAFs which overexpress CPT-1 with etomoxir obviously inhibits migration and invasion *in vitro* and decreases tumor growth and intraperitoneal dissemination *in vivo* 57.

Furthermore, it has been confirmed that adipocytes in TME affected by tumor-secreted factors leading to activated phenotypes are called cancer-associated adipocytes (CAAs) 58. Recently, emerging studies have underlined the bilateral interactions based on adipokines and lipids, between CAAs and tumors as well as their role in tumor development, which is also considered as part of a vicious circle 59. Researchers have demonstrated that, upon prolonged coculture with breast cancer cells, adipocytes loss almost all LDs, resulting in morphological changes toward a fibroblast-like phenotype, suggesting that FFAs could be released from these cells and transferred to cancer cells 60. The presence of CAAs may reprogram the metabolic activity of cancer cells from glycolysis to lipid-dependent energy production 61. Thus, after the initial interaction with lipid-loaded CAAs, tumor cells further liberate FFAs for the sake of maintaining invasive activity, which will be crucial for circulating cancer cells to reach distant organs 62.

## Lipid metabolism remodeling induction by extrinsic and intrinsic factors

It has been known that cancer cells are able to alter their metabolic state due to various intrinsic or extrinsic factors. Lipid metabolism reprogramming may occur in cancer cells as a result of intrinsic alterations like oncogenic mutations stimulate signal transduction components that either directly increases metabolic enzyme activity or transcription factors, thereby increasing expression of metabolic regulators 21.

Increased biosynthesis and uptake of lipids are supported by enhanced expression of the enzymes belonging to these pathways, which are regulated by a subclass of transcription factors called sterol regulatory-element binding proteins (SREBPs) 63. Several studies have found that SREBPs are activated downstream of the oncogenic signaling pathways, primarily at the PI3K/AKT/mTORC1 and RAS/ERK/mTORC1 signaling axis 64. The Myc oncogene and mutations of p53 tumor suppressor activate SREBP1 and SREBP2 respectively, also functioning as transcriptional coactivators for SREBP1 and SREBP2 to stimulate lipid synthesis 65, 66.

In fact, the physical and chemical nature of the TME may alter cellular lipid metabolism as a kind of extrinsic factor. Recently a study has reported that chronic acidosis in TME induces LDs formation in cancer cells and leads to more metastasis, with CD36 and diacylglycerol acyltransferase (DGAT) as key players to mediate LD biogenesis via the uptake of exogeneous FAs and triglyceride synthesis in a TGFβ2-dependent way 19. Also, it was demonstrated that hypoxia in cancer tissue stabilizes hypoxia-inducible factor-1α (HIF-1α), which induces fatty acid uptake and lipid storage in cells, contributing to cell growth and survival 67.

Growing evidence suggests that systemic factors and their intracellular pathways may activate oncogenic signals to reshape the metabolism in cancer cells 68. And these external signals fine-tune the cellular metabolism according to the availability of metabolites and the needs of the cell 21. As one of the metabolic diseases, obesity generates risk factors for cancers have been identified including insulin/insulin-like growth factor (IGF) axis, adipokines and cytokines 69. On the one hand, it was found that certain FFAs can be absorbed into cells in a CD36-dependent manner, resulting in the activation of mTOR to induce metabolic rewiring in obese-associated breast cancer cells 70. For another, obesity tends to contribute to the development of insulin resistance (IR). Despite the fact that insulin stimulates lipogenesis and inhibits lipolysis via activating the PI3K/Akt/mTORC pathway under normal circumstances, it has been reported that in insulin-resistant states of obesity and type 2 diabetes, hepatic lipid production is increased in concert with increased hepatic glucose production, which indicate that lipogenesis persists in IR as well 71, 72. Although signaling is impaired during IR conditions, mTORC1 which lies downstream of Akt still mediates SREBP-1c induction and function independently of Akt, therefore, lipogenic gene transcription is accelerated while glucogenic gene transcription remains alive 72.

Actually, it has been supposed that obesity and other metabolic diseases are associated closely with the gut microbiota which has been has considered as “metabolic organ”, plays a pivotal role in maintaining homeostasis of host lipid metabolism 73, 74. Compared with conventionally raised mice, germ-free (GF) mice fed with high-fat diet are resistant to diet-induced obesity and increased lipid metabolism. In addition, conventionalization of GF mice with normal mouse microbiota is able to promote *de novo* hepatic lipogenesis, indicating an unneglectable role of gut microbiota in altering metabolic response 75.

On the one hand, the human gut is regarded as reservoir of lipopolysaccharide (LPS) which consists mainly of outer membrane of gram-negative bacteria, and it has been reported that the enhanced LPS levels is related to increased adipose macrophage infiltration and IR 76. After LPS administration given to mice overnight, researchers found that the main proteins transporting FAs, CD36 and fatty acid transporter protein 4 (FATP4) decreased in response to LPS, suggesting that LPS inhibits FA absorption *in vivo*. And the effect of LPS on lipid metabolism was then demonstrated to be resulted from LPS stimulation induced overexpressed TNF-α in macrophages as TNF-α was able to activate caspase-3 which downregulates the expression levels of CD36 and FATP4 77. On the another hand, microbial processing of bile acid is supposed to result in a more hydrophilic bile acid pool and promotes excretion in the feces, while the bile acids lost in the fecal excretion also facilitates *de novo* synthesis from cholesterol 78. Also, it has been confirmed that gut microbiota is able to regulate the accumulation of hepatic natural killer T cells (NKT cells) and antitumor immunity in the liver to protect against primary and metastatic tumor by utilizing bile acid as messengers 79.

## Prospective

Specific molecular features of cancers that distinguish from the normal ones were called “hallmarks of cancer” and it is supposed that metabolism reprogramming has been considered one of the hallmarks of cancer 80. Of note, increasing studies have pointed out that a metabolic regulation of tumor progression affects many phenotypic traits of malignancy, including metastasis 81. It has been widely recognized that cancer cells prioritize aerobic glycolysis (Warburg effect) as the main energy source and convert excess pyruvate to lactate independently from oxygen availability 82. Actually, in addition to glycolysis, tumor cells can be expected to rewire their energy metabolism towards increased lipid synthesis to overcome therapy, an adaptation that further confers a more aggressive phenotype 83.

The so-called lipid metabolism remodeling in cells actually involves a series of altered enzyme activities which are likely to be associated with metastasis of various cancers 12. In lipid metabolism, FA metabolism including synthesis, uptake, further processing and catabolism plays an important role in controlling cancer progression 40, 84-86. Apart from FAs, it has been found that liver colonization of metastatic CRC cells required cholesterol biosynthesis pathways which involves key gene *SREBP2* and its downstream target gene *HMGCS* and *HMGCR,* and knocking down *SREBP2* significantly inhibited cancer liver metastasis 87. While, in breast cancer, decreased intracellular cholesterol was related to metastatic behavior of advanced stage of tumor cells and low expression of SREBP2 and 3-hydroxy-3-methylglutaryl coenzyme A reductase (HMGCR) evidently was associated with high EMT of the primary tumors 88. These data indicate that also as an important component of lipids, the role played by metabolism of cholesterol in cancer metastasis is still controversial and warrants further research.

In recent years, the inhibitors targeting major participants in lipid metabolism such as fatty acids synthesis, FAO, and cholesterol metabolism have been broadly tested in cancer treatment 89. As described in above content, CD36 is closely associated with tumor progression and metastatic initiation, based on which plenty of numerous studies and clinical trials targeting CD36 are being performed 90. In a mouse model of advanced stage epithelial ovarian cancer (EOC), pretreatment of thrombospondin-1 type I repeats (3TSR) which binds to the receptor CD36 could result in tumor regression, normalized tumor vasculature, and improved uptake of chemotherapy drugs 91. Besides, researchers have demonstrated that statins which are HMGCR inhibitors and previously used to diminish low-density lipoprotein cholesterol are also able to decrease the risk of metastatic PC and PC mortality 92.

Furthermore, exploring the factors influencing lipid metabolism remodeling may help find out new auxiliary therapeutic targets for cancer. On the one hand, it is thought that the metabolic demands of tumor cells were enhanced due to the mutational alterations in oncogenes and functional loss of onco-suppressors 93. As mentioned in previous section, oncogenic mutations stimulate signal transduction components which can directly increase transcription factors SREBPs, in turn increase expression of metabolic regulators. Fatostatin which is a recently discovered SREBP chemical inhibitor was found to downregulate the expression of SREBP-regulated enzymes for lipid metabolism, and decrease prostate tumor growth as well as distant lymph node metastasis 94. On the other hand, it is noteworthy that complexity of the metastatic process to some extent comes from interplays between cellular populations with various metabolic phenotypes within a given tumor, leading to metabolic symbiosis 95. TME is an assemble of tumor cells, fibroblasts, endothelial cells, immune cells, and other stromal cells recruited by tumor cells within the surrounding extracellular matrix 17. Resulting from the accelerated metabolism of cancer cells and cancer-associated cells, the hypoxia and acidosic characteristic of TME in turn reprogram the metabolism occurs in cancer and non-cancer cells, forming a pro-tumorigenic milieu around tumor cells 17, 96. Possessing the ability to decrease tumor acidity, the proton-pump inhibitor (PPI) such as esomeprazole was found to inhibit tumor growth in mouse models and improve acid-related chemoresistance 97. Interestingly, another PPI omeprazole was shown to block the FASN and dose-dependently suppress breast cancer cell metastasis 33.

To sum up, we review aberrant lipid metabolism occurring in cancer cells which plays a role in cancer metastasis. We also describe lipid metabolism alterations of surrounding cells in TME, which further contribute to cancer cell EMT, migration invasion and metastasis. This review can shed some light on the mechanisms underlying lipid metabolic reprogramming during tumor metastasis and reveal the intrinsic and extrinsic factors influencing the lipid metabolism reprogramming, and ultimately result in the identification of new therapeutic targets for cancer metastasis and improvement of patients' prognosis.

## Figures and Tables

**Figure 1:**
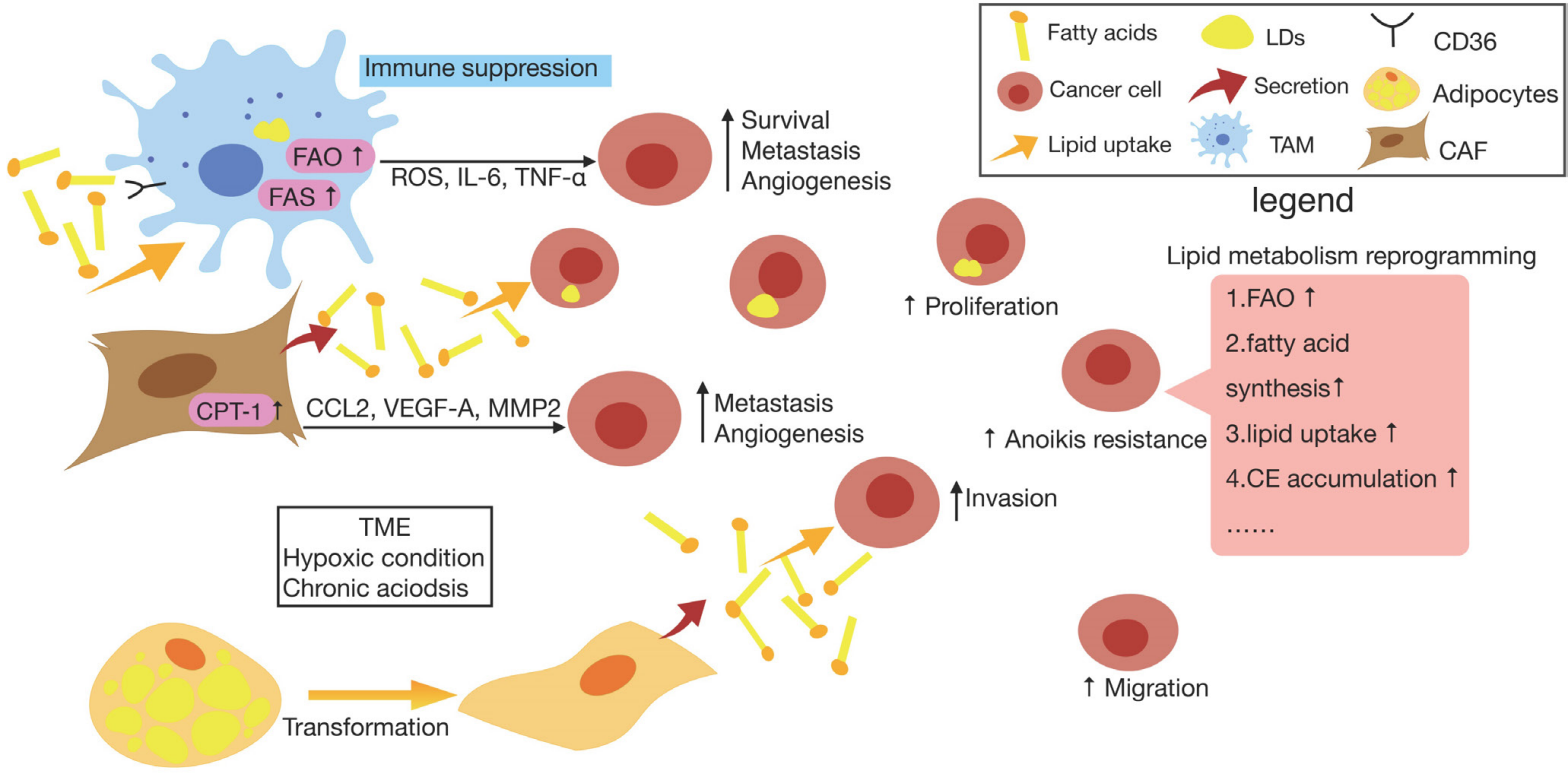
** Lipid metabolism reprogramming of cancer cells and surrounding cells in TME.** In cancer cells, there is an increase in lipid metabolism such as fatty acid synthesis (FAS), fatty acid oxygen (FAO), lipid uptake, cholesteryl easters (CE) accumulation etc., which promotes cancer cell proliferation, migration, anoikis resistance and so on. Also, resulting from the accelerated metabolism of cancer cells and cancer-associated cells, the hypoxia and acidosic characteristic of tumor microenvironment (TME) in turn reprogram the metabolism occurs in cancer cells and non-cancer cells. When exposed to chronic acidosis in TME, cancer cells may accumulate fatty acids (FAs) within lipid droplets (LDs) to reduce lipotoxicity. Furthermore, tumor-associated macrophage (TAM) tends to have a higher level of lipid uptake and accumulation via CD36, which accordingly promotes the FAO of TAMs to produce more energy. It also upregulates the lipid biosynthesis to generate more reactive oxygen species (ROS) and produce higher levels of extracellular cytokines like interleukin-6 (IL-6), tumor necrosis factor-α (TNF-α) and so on, which ultimately promotes cancer cell survival, metastasis, angiogenesis and immune suppression. As for cancer-associated fibroblast (CAF), it has been found that CAF can secrete FAs that is took up by cancer cells for the synthesis of other lipids, also CAF is able to enhance the expression of CPT-1A to actively oxidize FAs, thus enhancing the ability of CAFs to secrete cytokines such as CCL2, vascular endothelial growth factor-A (VEGF-A) and matrix metalloproteinases 2 (MMP2) which are associated with metastasis and angiogenesis of tumor cells. In addition, upon prolonged exposure to cancer cells, adipocyte losses almost all LDs, resulting in morphological changes toward a fibroblast-like phenotype and loads cancer cells with lipids, which contributes to maintaining invasive activity of cancer cells.

**Figure 2:**
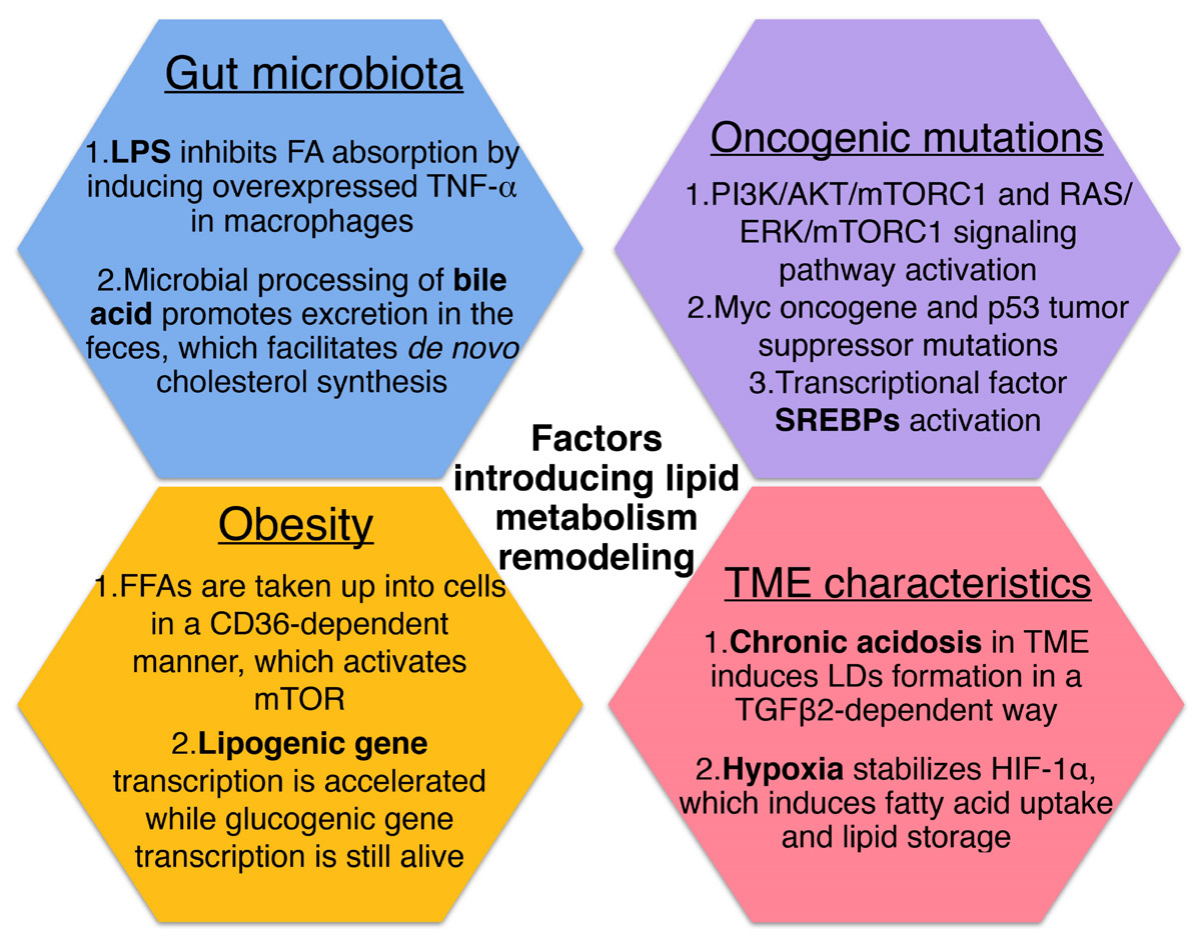
Factors that introduce cellular lipid metabolism remodeling.

**Table 1:** Lipid metabolism remodeling occurring in cancer cells and cancer related cells.

Cells	Lipid metabolism	Effects
Cancer cells	lipogenesis	CD44↑, anoikis resistance
	Lipid uptake	Initiate metastasis
	FAO	Maintain redox homeostasis
	Cholesterol esterification	Keep signaling pathway active
		
TAMs	Lipid uptake	immunosuppression
	Lipid biosynthesis	IL-6↑, TNF-α↑, ROS↑
		
CAFs	Synthesis/catabolism↑	Secrete FAs
	Lipid oxidation	CCL2↑, VEGF-A↑, MMP2↑
		
CAAs	Release FFAs	Reprogram the metabolism of cancer cells

CAAs, cancer-associated adipocytes; CAFs, cancer-associated fibroblasts; FAO, fatty acid oxidation; IL-6, interleukin-6; MMP2, matrix metalloproteinases 2; ROS, reactive oxygen species; TAM, tumor-associated macrophage; TNF-α, tumor necrosis factor-α; VEGF-A, vascular endothelial growth factor-A

## Abbreviations

ACAT: acyl-CoA cholesterol acyltransferase
ACC: Acetyl-CoA Carboxylase
ACLY: ATP citrate lyase
BC: breast cancer
CAAs: cancer-associated adipocytes
CAFs: cancer-associated fibroblasts
CE: cholesteryl easters
CPT: carnitine palmitoyl transferase
CRC: colorectal cancer
CSC: cancer stem cell
DGAT: diacylglycerol acyltransferase
EMT: epithelial-mesenchymal transition
FAs: fatty acids
FAO: fatty acid oxidation
FASN: fatty acid synthase
FATP4: fatty acid transporter protein 4
FFAs: free FAs
GF: germ-free
HCC: hepatocellular cancer
HIF-1α: hypoxia-inducible factor-1α
HMGCR: 3-hydroxy-3-methylglutaryl-CoA-reductase
IGF: insulin-like growth factor
IL-6: interleukin-6
IR: insulin resistance
MDSCs: myeloid-derived suppressor cells
MIC: metastasis-initiating cells
MMP: matrix metalloproteinases
NKT cell: natural killer T cell
LDs: lipid droplets
LN: lymph node
LPS: lipopolysaccharide
PC: prostate cancer
ROS: reactive oxygen species
SPF: specific pathogen-free
SREBPs: sterol regulatory-element binding proteins
TAM: tumor-associated microphage
TICs: tumor-initiating cells
TME: tumor environment
TNF-α: tumor necrosis factor-α
